# Estimation of Soil Moisture Content from the Spectral Reflectance of Bare Soils in the 0.4–2.5 μm Domain

**DOI:** 10.3390/s150203262

**Published:** 2015-02-02

**Authors:** Sophie Fabre, Xavier Briottet, Audrey Lesaignoux

**Affiliations:** Onera, BP74025 2 Avenue Edouard Belin FR-31055 Toulouse Cedex 4, France; E-Mails: xavier.briottet@onera.fr (X.B.); alesaignoux@gmail.com (A.L.)

**Keywords:** soil moisture content, bare soil, spectral reflectance, spectral indices, soil model

## Abstract

This work aims to compare the performance of new methods to estimate the Soil Moisture Content (SMC) of bare soils from their spectral signatures in the reflective domain (0.4–2.5 μm) in comparison with widely used spectral indices like Normalized Soil Moisture Index (NSMI) and Water Index SOIL (WISOIL). Indeed, these reference spectral indices use wavelengths located in the water vapour absorption bands and their performance are thus very sensitive to the quality of the atmospheric compensation. To reduce these limitations, two new spectral indices are proposed which wavelengths are defined using the determination matrix tool by taking into account the atmospheric transmission: Normalized Index of Nswir domain for Smc estimatiOn from Linear correlation (NINSOL) and Normalized Index of Nswir domain for Smc estimatiOn from Non linear correlation (NINSON). These spectral indices are completed by two new methods based on the global shape of the soil spectral signatures. These methods are the Inverse Soil semi-Empirical Reflectance model (ISER), using the inversion of an existing empirical soil model simulating the soil spectral reflectance according to soil moisture content for a given soil class, and the convex envelope model, linking the area between the envelope and the spectral signature to the SMC. All these methods are compared using a reference database built with 32 soil samples and composed of 190 spectral signatures with five or six soil moisture contents. Half of the database is used for the calibration stage and the remaining to evaluate the performance of the SMC estimation methods. The results show that the four new methods lead to similar or better performance than the one obtained by the reference indices. The RMSE is ranging from 3.8% to 6.2% and the coefficient of determination R^2^ varies between 0.74 and 0.91 with the best performance obtained with the ISER model. In a second step, simulated spectral radiances at the sensor level are used to analyse the sensitivity of these methods to the sensor spectral resolution and the water vapour content knowledge. The spectral signatures of the database are then used to simulate the signal at the top of atmosphere with a radiative transfer model and to compute the integrated incident signal representing the spectral radiance measurements of the HYMAP airborne hyperspectral instrument. The sensor radiances are then corrected from the atmosphere by an atmospheric compensation tool to retrieve the surface reflectances. The SMC estimation methods are then applied on the retrieve spectral reflectances. The adaptation of the spectral index wavelengths to the HyMap sensor spectral bands and the application of the convex envelope and ISER models to boarder spectral bands lead to an error on the SMC estimation. The best performance is then obtained with the ISER model (RMSE of 2.9% and R^2^ of 0.96) while the four other methods lead to quite similar RMSE (from 6.4% to 7.8%) and R^2^ (between 0.79 and 0.83) values. In the atmosphere compensation processing, an error on the water vapour content is introduced. The most robust methods to water vapour content variations are WISOIL, NINSON, NINSOL and ISER model. The convex envelope model and NSMI index require an accurate estimation of the water vapour content in the atmosphere.

## 1. Introduction

Surface soil moisture plays a key role to understand the exchange of water and heat energy between the land surface and the atmosphere through evaporation, to evaluate soil trafficability [1], to characterize plant health [2] or soil texture features like soil organic carbon or clay contents [3,4].

Remote sensing techniques have several advantages in comparison with others *in situ* methods (gravimetric, electromagnetic, thermal…) for monitoring Soil Moisture Content (SMC) [5], as they provide better temporal and spatial coverages [6]. At high spatial resolution, the most popular technique to sense soil moisture is based on active microwave sensor. This is due to the high sensitivity of the backscattered signal to the dielectric constant of soil and of its moisture [7] completed to its soil penetration capability. Nevertheless, the quality of the retrieved soil moisture is highly dependent on the surface roughness [7]. On the other side, hyperspectral imagery has demonstrated its potential to retrieve the soil moisture but its performance depends on the soil color and texture, the presence of organic material and crusts [7–10]. Further, the penetration depth in the optical domain is significantly lower and can only allow us to quantify the uppermost layer in a soil column.

Despite these drawbacks, there is a real interest to estimate the SMC from such sensors [11,12] as several hyperspectral space missions are planned for the near future: Hyperspectral Precursor of the Application Mission (PRISMA) [13], and Environmental Mapping and Analysis Program (EnMAP) [14] will be respectively launched in 2015 and 2018, or HYPerspectral IMagerie (HYPXIM) [15] planned before 2025. These three missions cover the reflective domain from 0.4 μm to 2.5 μm with a 10 nm spectral resolution. Such characteristics open the way to retrieve SMC from space data as proposed by [16].

Numerous studies have been conducted, mainly with laboratory measurements, to characterize the influence of SMC on the spectral reflectance. Angström [17] demonstrated through laboratory measurements that soil moisture content had an impact on the behavior of soil spectral reflectances in the solar domain. This work exhibited a decrease of the reflectance level as SMC increased due to a darkening of the soil surface. Later, other laboratory results over bare soils [18] confirmed this behavior which has, then, been used to develop SMC approaches based on spectral reflectances.

Nevertheless, investigations to explore the possibility of estimating soil moisture content from multispectral or hyperspectral remote sensing data, were penalized by the lack of specific databases. Liu *et al.* [19] measured the spectral reflectances of ten soil samples controlling the SMC during a drying process. These measurements have been completed using the database described in [20] including the spectral reflectances of about thirty natural soil samples in (0.4–2.5 μm) depending on the SMC and measured in the laboratory. This database was used to analyze the relationship between the SMC and the reflectance spectra. The laboratory measurements were limited to a few SMC levels (five or six) due to experimental constraints (drying time for example). In order to go beyond this limitation, a semi-empirical soil model was then proposed in [20] to simulate bare soil spectral reflectances for SMC levels not determined by experimentation and compared to state-of-the-art models. The conclusions pointed out that a representative database is necessary to analyze the impact of the SMC variation on soil spectral reflectances, to model this spectral behavior according to SMC and then to specify robust SMC assessment methods based on spectral signatures in the (0.4–2.5 μm) domain.

Three main approaches of SMC estimation can be distinguished: combination of spectral bands [21–23], exponential or Gaussian spectral models [24,25], and geostatistical methods [26–28]. Liu *et al.* [22] tested the first type of approaches using eighteen bare soil samples with different moisture contents characterized in the laboratory. Several analytical methods were tested: a relative approach normalizing the spectral reflectance of wet soil by the spectrum of the corresponding dry soil, a derivative approach based on the finite difference method, and an approach by difference computing a waveband difference. They concluded that SMC estimation using the relative method in the Short Wavelength InfraRed (SWIR) domain (1.4–2.5 μm) was more efficient. Concerning the use of spectral indices for estimating SMC, the best results were obtained with Water Index SOIL (WISOIL) [6], Shortwave Angle Slope Index (SASI) [23] and Normalized Soil Moisture Index (NSMI) [21]. These indices have been validated by reflectance measurements in the laboratory at different SMC over many bare soils. These results have led us to keep NSMI and WISOIL as reference methods. The main drawback of these indices is the use of wavelengths located in the water vapour absorption bands, making them very sensitive to the quality of the atmosphere compensation. Lobell *et al.* [25] have developed a spectral exponential model and applied it on four bare soil spectra measured in laboratory. Their results confirmed the strong potential of the SWIR domain for SMC estimation. Whiting *et al.* [24] proposed the Soil Moisture Gaussian Model (SMGM) to fit an inverted Gaussian function to the convex hull boundary points in the (1.8–2.8 μm) region of a bare soil spectrum. The area of one side of the Gaussian function above the spectral continuum was then related to SMC. The model performance estimated in laboratory measurements were: R^2^ of 94% and a mean RMSE of 0.027 g·g^−1^ [24]. However, the SMGM displayed poor performance for high soil water contents (like contents higher than 0.32 g·g^−1^). These models need *a priori* knowledge of the soil texture or have to be calibrated previously on soil samples taken from the analyzed area before the processing of the entire area. The geostatistical methods are based on the knowledge of the spatial distribution of soil moisture for predicting runoff at the observation area scale. These methods require *in situ* humidity measurements and use interpolation techniques for the analysis of the distribution of the soil moisture at spatial scale [26–28]. The most common interpolation techniques are moving average, trend surface and kriging. The applicability of these techniques depends on various factors such as distribution of sampled data in the observation area, the type of surfaces to be generated and tolerance of estimation errors. An adequate geometric correction processing in order to limit the high influence of topography in soil moisture estimation is necessary for the geostatistical methods. To conclude, there is a real need to specify methods undisturbed by the atmosphere compensation for an application on outdoor spectral measurements, without previous calibration on laboratory measurements of specific samples collected on the analyzed area, without any need for *in situ* SMC measurements for the method calibration, and reliable for extreme SMC values (like fully saturated soil or arid ground).

The objective of this work is then to present new methods of SMC estimation based on the spectral signature of bare soils measured in laboratory trying to overcome some limitations of the existing methods and analyze the impact of the atmosphere in order to anticipate their application on in field measurements and airborne hyperspectral acquisitions. Two approaches are then investigated to reach this objective: the spectral indices and the general spectral shape methods. The proposed methods are compared to the reference indices, NSMI and WISOIL. Section 2 presents the database of the laboratory measurements and the soil spectral model. Section 3 describes the SMC estimation methods. Section 4 compares the performance of the methods applied on the database and on simulated data representing at sensor level signal in order to analyze the impact of the sensor spectral resolution and the atmospheric water vapor content.

## 2. Description of the Reference Data Set and the Related Soil Spectral Model

As one of the proposed methods, the ISER model is based on an empirical spectral reflectance model depending on the SMC presented in [20], its main characteristics are presented in this section.

### 2.1. The Reference Database

The database [20] is composed of 32 natural soil samples, covering different ranges of texture (clay, limestone, sandy) and coloration. These samples were collected over eight locations in France (from South-West to South-East) (Table 1).

### 2.2. Measurement Method and Laboratory Devices

Soil spectral reflectances were measured in the laboratory with an ASD (Analytical Spectral Devices) Fieldspec-Pro spectroradiometer in the (0.4–2.5 μm) spectral domain. with a spectral resolution of 3 nm in the (0.4–1.0 μm) domain and of 10–12 nm in the (1.0–2.5 μm) domain. A Spectralon panel was used as white reference. The experimental protocol was as follows: each soil sample was put in a Petri dish (6 cm of diameter by 1 cm of thickness) and humidified until saturation.

**Figure 1.**
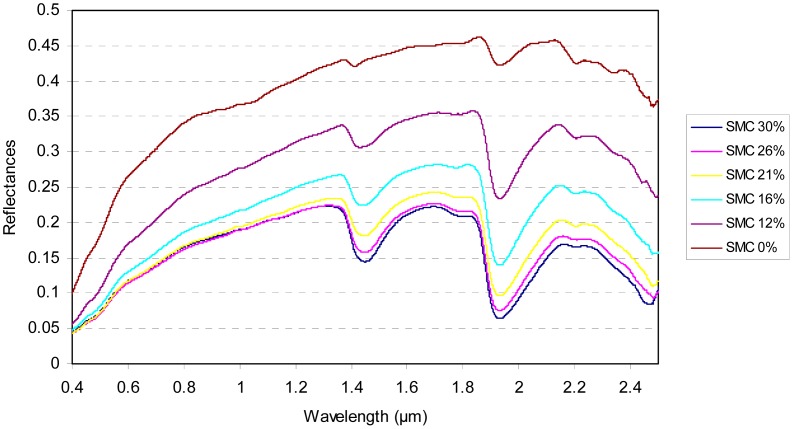
Spectral signatures according to SMC (in %g) for a soil sample of the area name 30Camargue (Table 1).

Successive SMC levels were artificially obtained by progressively drying the sample in a laboratory oven (at 333.15 °K during 30 min). After each drying step, the reflectance spectrum of each soil sample was measured and the gravimetric or mass SMC was measured by weighting the sample. The fully dried sample was obtained after a period of 24 h in the oven. Finally, the spectral reflectances of each soil sample were acquired for five or six SMC levels: six SMC levels for 30 soil samples and five SMC values for two soil samples. An illustration of the soil spectral reflectances for six soil moisture contents is given in Figure 1.

### 2.3. Description of the Soil Empirical Spectral Model

**Figure 2.**
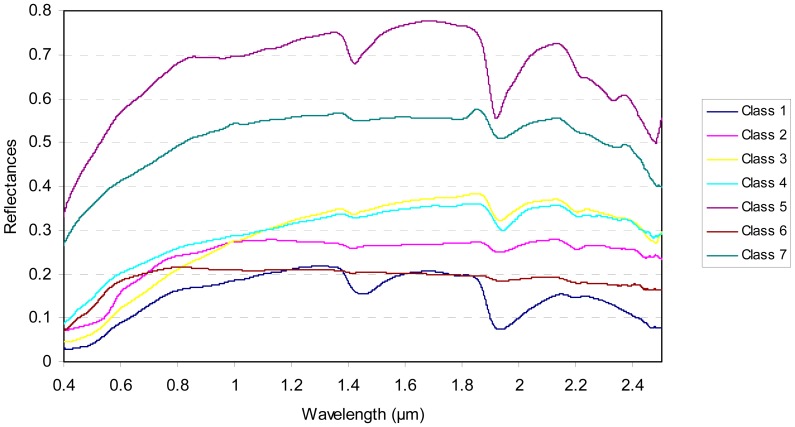
*A priori* soil classes according to the spectral behaviour of the dry soil samples.

The knowledge of the soil texture is assumed by most of the spectral soil models. To avoid this limitation, the proposed model is based on an *a priori* soil classification defined according to the global spectral shape of the dry soil reflectances [20]. Figure 2 illustrates the observed spectral behaviour of the dry samples (defined in the Table 1) in the VISible (VIS; (0.4–0.8 μm)) and Near and Shortwave InfraRed (NSWIR; (0.8–2.5 μm)) spectral domains.

According to [20], the soil samples with the same spectral behaviour are then grouped together in seven *a priori* spectral classes defined by Figure 2 and Table 2.

The semi-empirical soil model linking the spectral reflectance to the SMC for a given *a priori* soil class defined by the Table 2, is retained. Its analytical formulation is the following:

(1) $$\rho_{SMC_{g}}^{l}\left(\lambda \right)=\left(\lambda \right)\cdot SMC_{g}^{2}+b_{l}\left(\lambda \right)\cdot SMC_{g}+c_{l}\left(\lambda \right)$$

where *l* designs the soil spectral class, *a*, *b* and *c* are the spectral coefficients of the polynomial function in the solar domain. The spectral coefficient *c* is equivalent to the spectral signature of the dry soil and has a major impact on the polynomial function. The other spectral coefficients *a* and *b* are relatively less important [20].

Its intrinsic performance has been estimated by computing the standard deviation σ and the coefficient of determination R^2^. The soil model performance shows that, among all of the *a priori* classes, R^2^ is better than 97% with σ lower than 0.01. Moreover the model performance leads to a better correlation (98%) than the one obtained with the Lobell's model (79%) [20,25].

## 3. Description of the Methods to Estimate the Soil Moisture Content

### 3.1. Spectral Indices

Specific wavebands, known for stretching and bending vibrations of water and mineral-hydroxyl bands, have been successfully used to predict moisture content. The strongest water absorption strengths (at 1.2, 1.4, and 1.9 μm) correspond also to spectral region where atmospheric water vapor absorbs, rendering the methods using these spectral bands ineffective or underperforming. The state-of-the-art indices are listed in Table 3.

**Figure 3.**
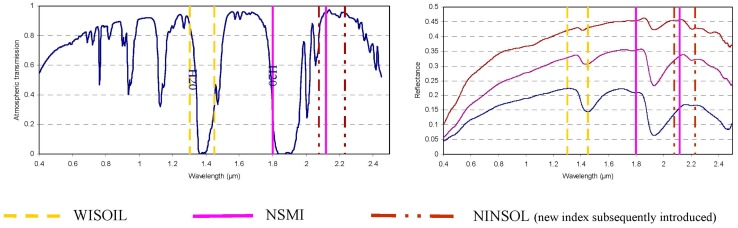
Location of the wavelengths operated by the spectral indices. These wavelengths are showed in the atmospheric transmission (**Left**) and the soil spectral signatures according to SMC (**Right**) graphs.

The wavelengths at 1.8 μm and 1.45 μm operated respectively by NSMI and WISOIL are located at the border of the atmospheric water vapour absorption band (Figure 3). The WISOIL and NSMI performance is then very dependent on the quality of the atmosphere compensation processing. Therefore, there is a need to define new spectral indices less sensitive to the atmosphere. Moreover it should be noted that WISOIL is using the 1.45 μm band corresponding to the atmospheric carbon dioxyd absorption band. Its impact is considered as negligible.

**Figure 4.**
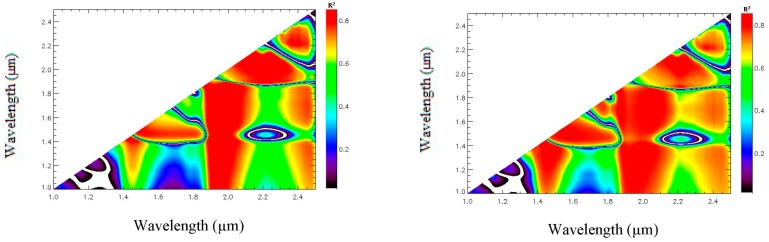
Determination matrix for *X_norm_*(*λ_i_*, *λ_j_*) computed with the reference database in the (1–2.5 μm) domain by linear regression (**Left**) and non linear regression (**Right**).

The new indices are defined according to the procedure proposed by Haubrock *et al.* [21]. The coefficient of determination for the linear regression [31] between SMC and a quantity *X_norm_*(*λ_i_*, *λ_j_*), derived from the spectral reflectance, is plotted in a matrix where the first wavelength value *λ_i_* is referred to by the abscissa axis and the second wavelength *λ_j_* is referred to by the ordinate axis (Equation (2) and Figure 4). This matrix is called regression matrix and the color scale from 0 to 0.87 represents the corresponding R^2^ value. This regression analysis is completed by a non-linear appropriate regression procedure [16,30].

According to this procedure, the normalized ratio *X_norm_*(*λ_i_*, *λ_j_*) defined by the following equation is computed on the data set described in the Section 2:

(2) $$X_{\text{norm}}\left(\lambda_{i},\lambda_{j}\right)=\frac{\rho \left(\lambda_{i}\right)-\rho \left(\lambda_{j}\right)}{\rho \left(\lambda_{i}\right)+\rho \left(\lambda_{j}\right)}$$

where *ρ*(*λ_i_*) and *ρ*(*λ_j_*) respectively represent the reflectance values at the wavelengths *λ_i_* and *λ_j_* belonging to the reflective domain (0.4–2.5 μm).

The corresponding regression matrix is shown on Figure 4. The wavelength pairs (*λ_i_*, *λ_j_*) which are very sensitive to SMC are located in the spectral range (1–2.5 μm). The highest R*^2^* coefficients are around 80% for wavelengths located in the (2–2.4 μm) range. The wavelength pairs leading to the highest determination coefficients between the SMC and the quantity *X_norm_*(*λ_i_*, *λ_j_*) are then deduced by applying a threshold to the determination matrix in order to define the wavelengths used to construct these new indices:

○ 2.076 μm and 2.228 μm for *X_norm_*(*λ_i_*, *λ_j_*): *R^2^* = 87% − linear regression
○ 2.122 μm and 2.23 μm for *X_norm_*(*λ_i_*, *λ_j_*): *R^2^*= 87% − non-linear regression.

The impact of atmospheric water vapour absorption bands is taken into account to choose these wavelengths by excluding the selected wavelengths corresponding to an atmospheric transmission lesser than 0.8 (Figure 3). These results lead to the following spectral indices:

Normalized Index of NSWIR domain for Smc estimatiOn from Linear regression (NINSOL)

(3) $$\text{NINSIOL}=\frac{\rho_{2.076}-\rho_{2.23}}{\rho_{2.076}+\rho_{2.23}}$$

- Normalized Index of NSWIR domain for Smc estimatiOn from Non-linear regression (NINSON)

(4) $$\text{NINSIOL}=\frac{\rho_{2.122}-\rho_{2.23}}{\rho_{2.122}+\rho_{2.23}}$$

### 3.2. General Spectral Shape Methods

**Figure 5.**
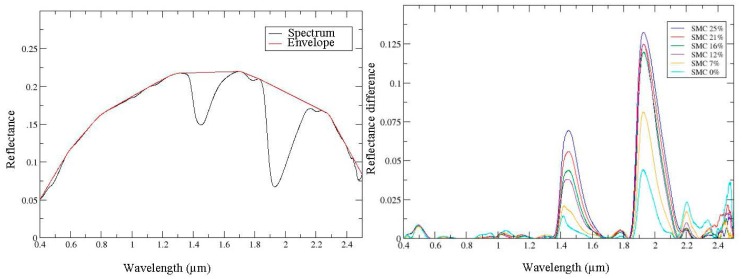
Illustration of the convex envelope method applied on a measured spectral signature (**Left**) and the difference between convex envelope and spectrum according to SMC (**Right**).

A new general spectral shape method based on the convex envelope and using the area criteria is proposed. The convex envelope of the spectral signature is computed on the entire solar spectrum (0.4–2.5 μm) (Figure 5, left). The area under the curve is achieved by subtracting the convex envelope and the spectrum itself. This criteria is related to the SMC (Figure 5, right). A linear regression model is then defined to link the SMC to this area.

A second method using the soil semi-empirical spectral model (presented in Section 2.3) is proposed. This model (Equation (1)) is inverted in order to retrieve, for a given *a priori* soil class and the related spectral signature, the corresponding SMC. The retrieved gravimetric soil moisture content SMC_g_ is the one that minimizes the quadratic error defined by the following equation:

(5) $$E=\sum_{i=0}^{q}{\left(\rho_{\text{input}}\left(i\right)-\left(a_{l}\left(i\right)\cdot SMC_{g}^{2}+b\left(i\right)\cdot SMC_{g}+c\left(i\right)\right)\right)}^{2}$$

where *i* is the wavelength, *q* is the number of wavelengths on the spectral range (0.4–2.5 μm) and *ρ_inpit_* is the spectral reflectance.

In the following, this method is named Inverse Soil semi-Empirical Reflectance (ISER) model.

## 4. Results and Discussion

### 4.1. Comparison of the SMC Estimation Methods

#### 4.1.1. Principle of the Performance Analysis

The SMC estimation methods described in the Section 3 are applied on the reference database defined in Section 2.1 in order to compare their performance using the coefficient of determination (R^2^) and the Root Mean Square Error (RMSE) [30]. The dataset is divided in two groups. The first one (called calibration data set) is used to calibrate the methods and includes 95 spectral signatures. The second one (called validation data set) is used to the validation and includes the 95 remaining spectral signatures. For each method, the comparison process is as follows:

- Calibration stage: The measured spectral signatures and the corresponding SMC of the calibration data set are used to achieve the linear regression between the index values and the SMC for NINSOL, WISOIL, NSMI and between the area values and the SMC for the convex envelope model. Similarly, the non-linear regression between SMC and the NINSOL values is deduced owing to the calibration data set;
- Validation stage: The SMC is estimated with the validation data set owing to the linear (or non- linear) relation deduced in the calibration stage. The quality of the SMC estimation is assessed by computing the R^2^ and the RMSE.

The RMSE is expressed as follows:

(6) $$\text{RMSE}=\sqrt{\frac{\overset{N}{\underset{i=1}{\text{∑}}}{\left(SMC_{i}^{\text{mes}}-SMC_{i}^{\text{est}}\right)}^{2}}{N}}$$

where ${\text{SMC}}_{i}^{\text{est}}$ is the estimated SMC for the soil sample *i*, ${\text{SMC}}_{i}^{\text{mes}}$ is the measured SMC for the same sample *i* and N is the number of samples.

**Figure 6.**
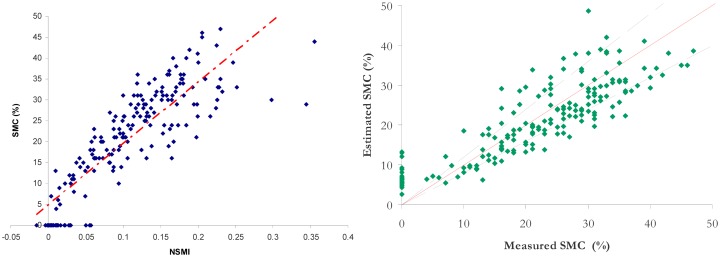
Variation of the SMC according to NSMI (calibration stage, **Left**) and representation of estimated SMC with NSMI according to measured SMC (validation stage, **Right**). The dashed black lines represent the boundaries of an error of 20%.

Figure 6 represents the calibration (left) and the validation (right) stages for NSMI.

#### 4.1.2. Results

The comparison between the estimated SMC by the ISER model with the validation data set and the measured SMC is illustrated for each *a priori* soil spectral class in the Table 4. The RMSE ranges between 2% and 4%. R^2^ is better than 0.90 according to the *a priori* soil class.

**Figure 7.**
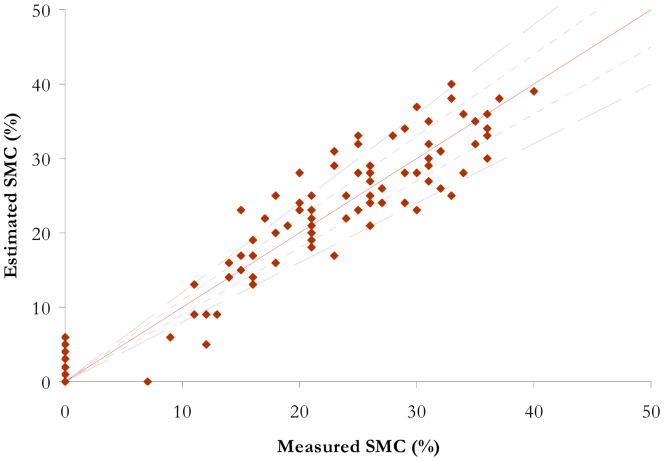
Representation of estimated SMC with the inverse model according to measured SMC for the *a priori* soil class 3. The dashed black lines represent the boundaries of an error of 20%.

The soil spectral classes composed by an unique soil (class 2, class 5 and class 7) have RMSE values lower than 3% and a corresponding error lower than 10%. Soil class 3, which includes the largest number of samples, is mainly characterized by an error on the SMC estimation lower than 20% (Figure 7).

**Figure 8.**
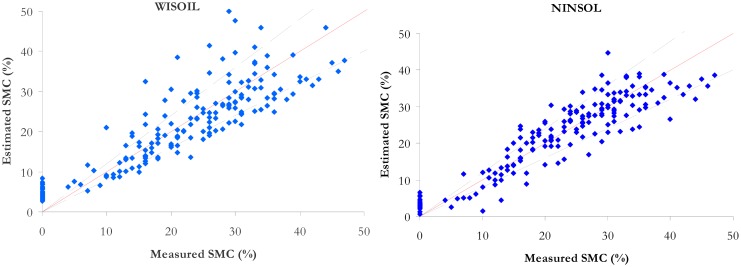
Representation of estimated SMC according to measured SMC for WISOIL (**Left**) and NINSOL (**Right**). The dashed black lines represent the boundaries of an error of 20%.

The synthesis of the performance comparison is given in Table 5. The RMSE and R^2^ are computed with the validation data set. This is equivalent for the ISER model to calculating the RMSE and R^2^ for all the classes. The results for NSMI, WISOIL and NINSON are illustrated in the Figure 6 (right) and the Figure 8. The R^2^ values of NSMI and WISOIL are respectively 73% and 79%. Better results are obtained with NINSOL (87%) and NINSON (85%). 52% of the SMC values obtained with NSMI have an error higher than 20% (24% for WISOIL). For the new indices, 70% of the estimated SMC values are characterized by an error lower than 20%. For the convex envelope model, R^2^ is equal to 0.8. The corresponding RMSE is 5.6%.

The R^2^ values obtained with NSMI and WISOIL are consistent with those in literature. In [21], NSMI led to R^2^ value of 0.61 for 467 field soil samples measured in the laboratory with their natural soil moisture rates and consisting of different sands and clayey substrates. A similar coefficient of determination (R^2^ = 0.6) was observed for 11 artificially prepared samples (each sample was artificially wetted in steps of 2% until saturation which ranged between 18% and 24% depending on the soil sample). In [6], the R^2^ values obtained with WISOIL ranged from 0.72 to 0.96 for 3 soil samples (a dark colored silt/loam soil with approximately 5% organic matter, a red colored clay soil and an iron rich soil) according to the SMC level.

The performance of the convex envelope is equivalent to WISOIL and NSMI performance. NINSON and NINSOL indices provide similar results. The inverse soil spectral model shows overall the best performance. All the methods, except for ISER, can be applied whatever the soil texture and without *a priori* information on the soil spectral behavior. The ISER model leads to the best performance with further information on the dry soil spectral signature.

### 4.2. Sensibility of the SMC Estimation Methods to the Atmosphere Conditions

#### 4.2.1. Method Description

In order to evaluate the sensitivity of the SMC estimation methods to the atmospheric water vapour content, end-to-end simulations are achieved. The spectral radiances at the top of the atmosphere are computed with a radiative transfer tool according to a given spectral signature of the database (see section 2.1). The spectral radiances are then integrated over the wavelength bands of the hyperspectral sensor. An atmospheric compensation tool is then applied to retrieve the on ground surface reflectances and to deduce the estimated SMC with the methods described in Section 3.

The radiative transfer computations are done with MODerate resolution atmospheric TRANsmission (MODTRAN4 [31,32]). The surface reflectance retrieval is achieved with the atmospheric compensation tool COde de Correction atmosphérique Hyperspectrale d'Images de Senseurs Embarqués (Cochise [33]) from spectral radiances acquired by a hyperspectral system on board an aircraft. Two types of errors are simulated:

- The atmosphere is assumed well known. Let US0 (called nominal case) be the standard atmosphere used to compute the spectral radiances at sensor level. The SMC estimation methods are then applied on the retrieved spectral reflectances with the atmospheric compensation tool. The adaptation of the spectral index wavelengths to the sensor spectral bands (band centre wavelengths and spectral resolution) and the application of the convex envelope and ISER models to boarder spectral bands lead to an error on the SMC estimation. The intrinsic error of the atmospheric compensation tool has an impact on the spectral signatures used to estimate the SMC (RMSE is within 3% in [34]). The SMC values estimated for US0 are compared to the SMC values computed with the SMC estimation methods applied on the database. The SMC values for US0 are then compared with the measured SMC of the database with the R^2^ and the RMSE;
- To achieve the atmosphere compensation, the atmosphere conditions need to be known. In this work, we only consider the most impacting factor for the SMC estimation methods: the water vapour content. An error is then introduced on the water vapour content. The error on the SMC due to an uncertainty on the water vapour content (represented by the atmosphere *USx*, *x* = 1, 2, 3 or 4) is expressed as a percentage:
  (7) $$\delta_{i}=\frac{SMC_{i}\left(US0\right)-SMC_{i}\left(\text{USx}\right)}{SMC_{i}\left(US0\right)}$$

where *i* is the soil sample, *SMC_i_*(*USx*) is the estimated SMC corresponding to the US*x* atmosphere (*x* = 1, 2, 3 or 4) for the sample *i*.

The corresponding bias is expressed in the SMC unit (%g):

(8) $$\text{bias}\left(\text{USx}\right)=\sum_{i=1}^{N}SMC_{i}\left(US0\right)-SMC_{i}\left(\text{USx}\right)$$

N represents the number of soil samples.

#### 4.2.2. Input Description

The scene is composed of a flat and homogeneous ground in a standard atmosphere (US Standard Atmosphere 1976) [32] corresponding to an integrated water vapour content of 1.5 g·cm^−2^. The ground spectral properties are defined owing to the database.

The hyperspectral sensor is the airborne HYMAP hyperspectral instrument [35] covering the spectral domain (0.4–2.5 μm) with 128 spectral bands, a spectral resolution ranging from 15 to 20 nm and a spatial resolution around 4 m at 2000 m height. The selected wavelengths (defined in the Section 3.1) associated to each index have first to be adapted to the spectral characteristics of the sensor. Nevertheless, the difference between the index wavelengths and the central wavelengths of the HYMAP spectral bands does not exceed 3 nm. The inputs of the US0 nominal case are summarized in Table 6.

**Figure 9.**
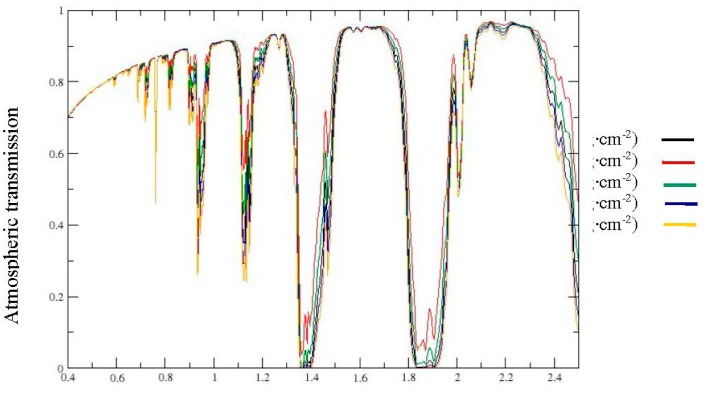
Total atmospheric transmission of the US standard profile for integrated vapour contents from 0.5 g·cm^−2^ to 2.5 g·cm^−2^.

The impact of the atmospheric water vapour content on the SMC estimation methods is analyzed owing to four simulation cases (US1 to US4) corresponding to the variation of the integrated vapour content from 0.5 g·cm^−2^ to 2.5 g·cm^−2^ (the water vapour content values are 0.5, 1.0, 2.0, 2.5 g·cm^−2^, 0.5 g·cm^−2^ corresponds to a very dry atmosphere and 2.5 g·cm^−2^ represents a tropical atmosphere). The variation of water vapour content modifies the total atmospheric transmission in depth and in width (Figure 9).

#### 4.2.3. Results

**Figure 10.**
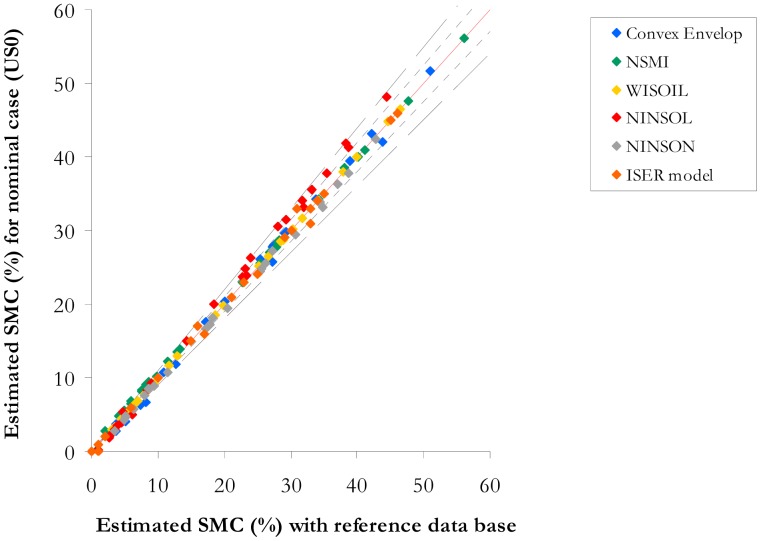
Spectral indices (NSMI, WISOIL, NINSOL, NINSON) and general shape methods (convex envelope, ISER model—Representation of the SMC computed on the simulated spectra (nominal case US0) according to SMC estimated on the corresponding measured spectra (point lines on each side of the central line give the limit of an error of 5% and the long-dashed lines an error of 10%).

The results are provided in Figure 10 and Table 7. The sensor spectral resolution has a limited impact on the spectral indices and the spectral shape methods performance (Figure 10). The NSMI relative error is around 1% for SMC higher than 15% and 10% for SMC lower than 15%. The WISOIL exhibits a relative error in the order of 1.5%. NINSOL offers a relative error lower than 10% for SMC over 15%. The NINSON relative error is about 5%. The relative error of the convex envelope model is 6%. Its performance is comparable to NSMI according to the SMC levels. The ISER model is characterized by a relative error lower than 10% whatever the SMC value. The highest RMSE value and lowest R^2^ are related to NSMI. The convex envelope area and WISOIL deliver similar performance as NINSOL and NINSON. The ISER model is the most robust.

Secondly, the impact of an uncertainty on the water vapour content is analysed. The results are presented in the Table 8. The NSMI errors are lower than 5% for SMC higher than 15% and lower than 10% for lower SMC values, regardless of the atmospheric water vapour content. For instance, when the water vapour content is increasing from 1.5 g·cm^−2^ to 2.5 g·cm^−2^ (US0 to US4), the average error of the SMC assessment is increasing of around 6 %. NSMI, operating with the Hymap spectral bands based on central wavelengths at 1.80 μm and 2.12 μm, is impacted by the water vapour content variation owing to the wavelength at 1.80 μm (Table 3). The NMSI leads to the higher bias values.

The variation of the water vapour absorption bandwidth has a low influence on WISOIL, NINSON and NINSOL owing to their operated wavelengths (Equations (3) and (4)). The average error on the SMC assessment and the bias are stable regardless of the error on the water vapour content (less than or equal to 1.3%).

**Figure 11.**
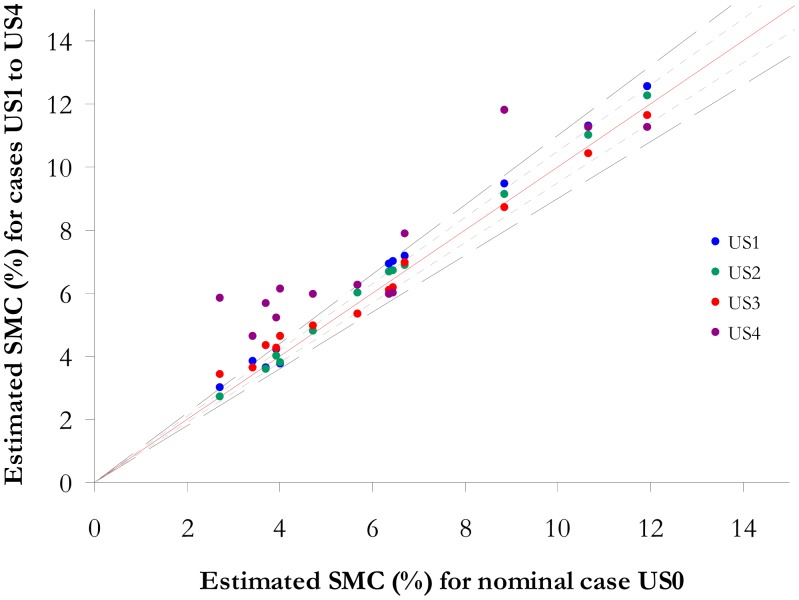
Convex envelope method—Representation of the SMC estimated for the US1 to US4 cases according to SMC estimated for the US0 case for SMC lower than 15%.

The estimated SMC with the convex envelope area leads to error lower than 5% for SMC values higher than 15%. Moreover, the increase of the water vapour content results in a significantly overestimation of the SMC value (if the water content increases from 1.5 g·cm^−2^ (US0) to 2.5 g·cm^−2^ (US4) for SMC values lower than 15% (negative values of bias in the Table 8 and Figure 11), To conclude, the overestimation of the atmospheric water vapour content has an important impact for this method. Thus, NSMI and the convex envelope lack robustness if the water vapour content is not well known. WISOIL, NINSOL, NINSON and the ISER model are less sensitive to the variation of this atmospheric parameters than NSMI and the convex envelope. The ISER model is the most robust to atmospheric water vapour content errors.

## 5. Conclusions

New SMC estimation methods based on soil spectral signatures of the reflective spectral domain (0.4–2.5 μm) are presented. These methods are validated on a data set including 32 different soil samples collected in the South of France. These methods have been compared to existing methods found in the literature, and their sensibility to the atmosphere compensation process has been analyzed.

The specification of SMC assessment criteria is put forth using existing reference data measured in the laboratory. Determination matrixes are used to define two spectral indices (NINSON and NINSOL) by taking into account the atmospheric transmission. These new indices are compared to the existing spectral indices NSMI and WISOIL. The proposed spectral shape methods include the convex envelope model and the ISER model based on the inversion of the empirical soil spectral model. This last one aims to estimate the SMC assuming that the soil spectral class (related to the spectral shape of the dry soil) is known *a priori*. All these methods are applied on the database in order to evaluate their performance. The NINSON and NINSOL indices provide similar performance close to the performance of WISOIL and NSMI. The ISER model shows overall the best performance.

The work is then extended to remote sensing. Synthetic airborne radiances are simulated using the spectral reflectances of the database. After a spectral integration to simulate the HYMAP spectral bands, an adaptation of the spectral index wavelengths to the sensor bands is necessary. The induced errors are then lower than 10% and the most robust and efficient method is the ISER model (RMSE of 3%, in comparison to 6%–8% for the others methods).

The atmospheric compensation tool is then applied by introducing an uncertainty on the water vapor content. The sensitivity study in relation to the atmospheric water vapor content has yielded the following results:

- Whatever the selected method and error, the most important impact is obtained for SMC lower than 15%;
- NSMI and the convex envelope model are less robust than the other methods;
- WISOIL, NINSOL and NINSON are slightly affected by the variation of the water vapor content;
- The ISER model exhibits the best performance.

In the near future, these methods will be tested on a wider data set including other soil textures. Further works will be pursued to evaluate the performance to retrieve the SMC from real remote sensing images acquired on a bare soil landscape in order to define rules for their use according to their robustness taking into account the soil roughness, the presence of sparse vegetation, the presence of soil organic carbonates … In the presence of sparse vegetation, a sensitivity study will be conducted in order to analyze the atmospheric carbon dioxide impact on the performance of WISOIL. The ISER model does not need the soil texture but rather uses as input the dry reflectance shape in order to deduce the corresponding spectral class. Multi-temporal analysis is an efficient opportunity to access to this information. The application of the ISER model to multi-temporal data will be tested in the future. For achieving these objectives, several airborne experiments are planned on soils with different textures at different periods in the year with joint *in situ* measurements.

## Figures and Tables

**Table 1.** Characteristics of the bare soil samples where the area name is defined as [France Department ID][Nearby Town], followed by the number of collected samples, the geographic area location and the corresponding Munsell color code (Y: Yellow; YR: Yellow Red). Extracted from [20].

**Area Name**	**Number**	**Geographic Location**		**Munsell Color Code [29]**		
	
		**Latitude**	**Longitude**	**Hue**	**Value**	**Chroma**
11Belvis	1	42°51′02″N	02°04′32″E	2.5Y	6	6
11Malves	1	43°15′08″N	02°26′26″E	2.5Y	7	4
12Vabres	1	43°41′35″N	02°25′35″E	2.5YR	4	4
13Crau	2	43°8′59″N	06°04′27″E	10YR	4	6
				10YR	5	4
24Coulouniex	1	45°11′11″N	00°42′00″E	2.5Y	4	2
30Camargue	18	43°40′37″N	04°37′43″E	2.5Y	5	2
				2.5Y	6	2
30Pujaut	1	44°00′17″N	04°46′29″E	2.5Y	8	1
31Fauga	2	43°23′47″N	01°17′39″E	2.5Y	4	3
31Lauraguais	2	43°23′59″N	01°43′05″E	2.5Y	5	4
				2.5Y	5	3
81Lautrec	1	43°42′22″N	02°08′20″E	2.5Y	3	3
81StJulien	1	43°59′22″N	02°20′45″E	5Y	8	1
84Avignon	1	43°56′55″N	04°48′30″E	2.5Y	7	2

**Table 2.** The *a priori* spectral classification of the dry soil samples.

***A Priori* Soil Spectral Class**	**Number of Soil Samples**	**Spectral Behaviour Description of Dry Soil Samples in the VIS and NSWIR Domains**
Class 1	5	VIS: Concave spectral signature, then convex without high level (presence of iron oxide) NSWIR: Concave spectral signature in the 0.8–1.2 μm range, then convex with a medium level
Class 2	1	VIS: Concave spectral signature then convex without high level NSWIR: Convex spectra in the 0.8–2.5 μm domain with a medium level
Class 3	18	VIS: Concave spectral signature, then convex without high level NSWIR: Spectra with slope ascending in the 0.8–1.2 μm domain, then convex with a medium level
Class 4	3	VIS: Spectral signature weakly concave with low level (presence of organic matter) NSWIR: Spectra with slope ascending in the 0.8–1.2 μm domain, then convex with a medium level
Class 5	1	VIS: Spectral signature not particularly convex with high level (large amount of calcium and small quantity of iron oxide) NSWIR: Concave spectral signature in the 0.8–1.2 μm range, then convex with a medium level
Class 6	2	VIS: Concave spectral signature, then convex without high level NSWIR: Weakly concave spectral signature in the 0.8–1.2 μm domain with an upslope and a low level
Class 7	1	VIS: Spectral signature not particularly convex with high level NSWIR: Convex spectra in the 0.8–2.5 μm domain with a medium level

**Table 3.** Existing spectral indices suitable for SMC assessment (ρ reflectance).

**Spectral Index**	**Specific Spectral Bands**	**Formulation**
NSMI	1.800 μm; 2.119 μm	$\frac{\rho_{1.8}-\rho_{2.119}}{\rho_{1.8}-\rho_{2.119}}$
WISOIL	1.30 μm; 1.45 μm	$\frac{\rho_{1.45}}{\rho_{1.30}}$

**Table 4.** The R^2^ and RMSE values obtained with the inverse model for each a priori soil class.

**Soil Spectral Class**	**R^2^**	**RMSE (%)**
Class 1	0.90	4.1
Class 2	0.95	2.2
Class 3	0.90	4.0
Class 4	0.95	3.6
Class 5	0.97	1.9
Class 6	0.91	3.7
Class 7	0.96	2.1

**Table 5.** Synthesis of methods and performance computed with validation data set.

**Method**	**Type**	**Reference**	**R^2^**	**RMSE (%)**
NSMI	Index	[21]	0.74	6.2
WISOIL	Index	[6]	0.79	5.7
NINSOL	Index	§ 3.1	0.87	4.4
NINSON	Index	§ 3.1	0.85	4.8
Convex envelope model	General shape	§ 3.2	0.80	5.6
ISER model	General shape	§ 3.2	0.91	3.8

**Table 6.** Parameters of the nominal case US0.

**Hyperspectral System**	**HYMAP**
Acquisition conditions	System altitude: 2000 m Viewing angle: Nadir Hour: 11 h UT
Atmosphere	Atmospheric profile: US Standard Integrated vapour content: 1.5 g·cm^−2^
Scene description	Surface temperature: 293 °K Measured spectral signature (one by soil spectral class) for three SMC levels varying from 0% to 46% corresponding to dry, intermediary and saturated levels

**Table 7.** Synthesis of the R^2^ and RMSE values computed on the estimated SMC for US0 and the corresponding measured SMC.

**Method**	**R^2^**	**RMSE (%)**
NSMI	0.79	7.8
WISOIL	0.83	6.7
NINSOL	0.80	6.3
NINSON	0.83	6.4
Convex envelope model	0.80	7.1
ISER model	0.96	2.9

**Table 8.** Synthesis of the relative error (mean, standard deviation) (Equation (7)) and bias values (Equation (8)) computed on the estimated SMC for US0 case and the corresponding SMC for other simulation cases.

**Method**	**Bias (%g)**				**Mean Relative Error (%)**				**Standard Deviation of the Relative Error (%)**			
			
**Case**	**US1**	**US2**	**US3**	**US4**	**US1**	**US2**	**US3**	**US4**	**US1**	**US2**	**US3**	**US4**
NSMI	0.5	0.3	−0.3	−0.6	4.8	2.6	2.8	5.8	4.2	2.3	2.5	5.1
WISOIL	−0.1	−0.1	0.1	0.1	0.7	0.3	0.3	0.7	0.8	0.4	0.4	0.8
NINSOL	−0.1	−0.1	0.1	0.1	1.1	0.5	0.5	1.1	2.5	1.1	1.1	2.4
NINSON	−0.1	−0.1	0.1	0.1	1.3	0.6	0.5	0.9	2.6	1.3	1.0	1.9
Convex envelope model	−0.6	−0.3	-0.1	−0.1	4.7	2.2	4.4	16.2	3.0	1.6	6.0	25.0
ISER model	0.	0.	0.1	0.1	0.0	0.0	0.1	0.5	0.0	0.0	0.6	1.9
